# A dendritic cell population responsible for transglutaminase 2–mediated gluten antigen presentation in celiac disease

**DOI:** 10.1172/jci.insight.196102

**Published:** 2025-08-28

**Authors:** Fu-Chen Yang, Harrison A. Besser, Hye Rin Chun, Megan Albertelli, Nielsen Q. Fernandez-Becker, Bana Jabri, Chaitan Khosla

**Affiliations:** 1 Department of Chemistry, Stanford University, Stanford, California, USA.; 2 Stanford Medical Scientist Training Program, Stanford University School of Medicine, Stanford, California, USA.; 3 Committee on Immunology, University of Chicago, Chicago, Illinois, USA.; 4 Department of Comparative Medicine, Stanford University School of Medicine, and; 5 Division of Gastroenterology and Hepatology, Department of Medicine, Stanford University, Stanford, California, USA.; 6 Department of Medicine, University of Chicago, Chicago, Illinois, USA.; 7 Department of Chemical Engineering and; 8 Sarafan ChEM-H, Stanford University, Stanford, California, USA.

**Keywords:** Autoimmunity, Gastroenterology, Antigen, Dendritic cells, MHC class 2

## Abstract

In celiac disease (CeD), a gluten-dependent autoimmune disorder, transglutaminase 2 (TG2), deamidates selected glutamine residues in gluten peptides, while HLA-DQ2 presents deamidated antigens to inflammatory T cells. The cellular sources of pathogenic TG2 and DQ2 are unclear. Using chemical biology tools, we show that intestinal CD103^+^ dendritic cells (DCs) couple cell-surface TG2 to the endocytic LRP1 receptor to simultaneously deamidate gluten antigens and concentrate them in lysosomes. In DQ2-transgenic mice, CD103^+^ DCs loaded with deamidated antigens migrate from intestinal lamina propria and Peyer’s patches into mesenteric lymph nodes, where they engage T cells. In turn, gluten antigen presentation upregulates intestinal TG2 activity. The tool (HB-230) used to establish a role of CD103^+^ DCs in gluten antigen presentation and TG2 activation in mice also revealed that the TG2/LRP1 pathway is active in human CD14^+^ monocytes. Within this population of circulating monocytes, a DC subset with the gut-homing β7-integrin marker is elevated in patients with CeD with active disease compared with nonceliac controls or patients on a gluten-free diet. Our findings not only inform the cellular basis for gluten toxicity in CeD, but they also highlight the immunologic role of an enigmatic protein of growing therapeutic relevance in CeD and other immune disorders.

## Graphical abstract

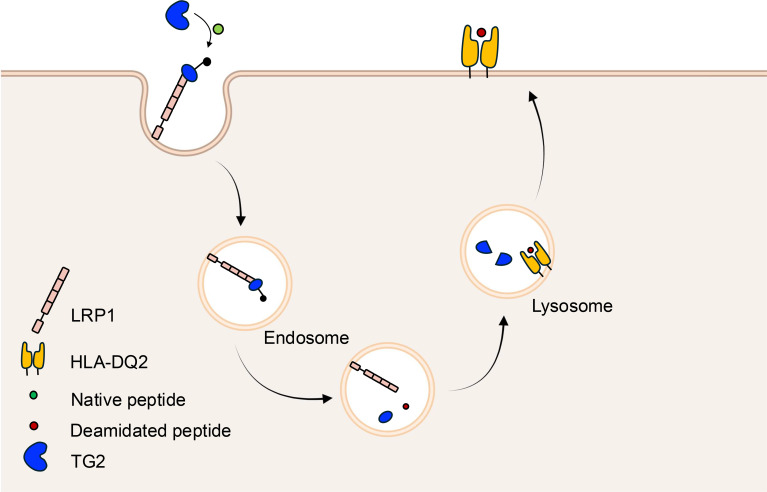

## Introduction

Celiac disease (CeD) is a chronic autoimmune disorder characterized by persistent inflammation of the small intestine that affects approximately 1 in 100 individuals worldwide. Notwithstanding advances in our understanding of disease pathobiology, the current management of CeD remains limited to a strict lifelong gluten-free diet (GFD). Therefore, identifying pharmacologically relevant mechanisms that underpin the onset and/or persistence of CeD remains a priority.

CeD is triggered in genetically predisposed individuals by the ingestion of gluten, a family of proteins found in wheat, barley, and rye. Partially digested gluten peptides are believed to cross the intestinal epithelium and be taken up by antigen-presenting cells (APCs) in the lamina propria below, where they are presented to gliadin-specific CD4^+^ T cells. Over 90% of individuals with CeD express human leukocyte antigen DQ2.5 (encoded by DQA1*0105 and DQB1*0102; hereafter referred to as HLA-DQ2), while a smaller proportion of patients express HLA-DQ8.

**Figure 1:**
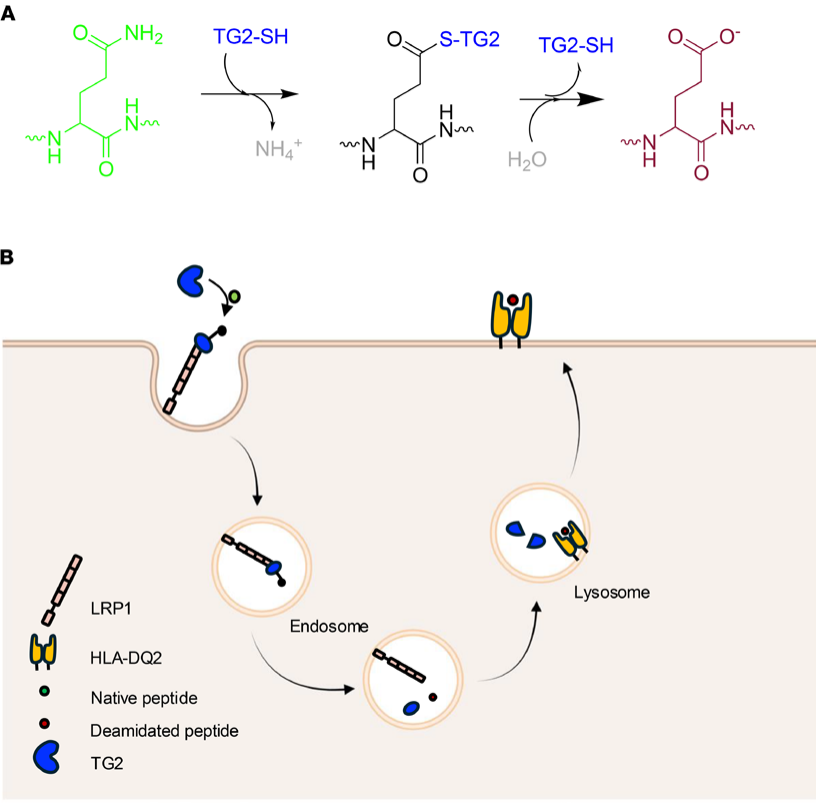
The TG2/LRP1 pathway allows simultaneous receptor-mediated endocytosis and deamidation of antigenic gluten peptides. (**A**) Transglutaminase 2 (TG2) catalyzes deamidation of selected Gln (Q) residues in its substrates via a 2-step mechanism. In the first half-reaction, the thiol (SH) functional group of a Cys residue in the active site of TG2 forms a covalent thioester intermediate with the reactive Gln residue, resulting in the release of an ammonium (NH_4_^+^) ion. The second half-reaction involves hydrolysis of this thioester intermediate resulting in a net conversion of the Gln residue into a Glu (E) residue. Alternatively, if a suitable primary amine is available, the thioester intermediate can undergo aminolysis in the second half-reaction, leading to formation of an isopeptide bond (not shown). (**B**) On the surface of certain antigen-presenting cells, catalytically active TG2 (blue) captures gluten antigens (green) as their corresponding thioester intermediates (black). However, before the thioester intermediate can undergo hydrolysis, it is recognized by the low-density lipoprotein-related protein 1 (LRP1) receptor on these antigen-presenting cells. Binding to LRP1 stabilizes the transient enzyme-substrate complex while activating receptor-mediated endocytosis. The TG2-bound thioester is released and hydrolyzed in the acidic environment of endosomes, leading to endosomal delivery of the deamidated gluten peptide (red). Once in the lysosome, the deamidated gluten peptide is loaded into the antigen binding pocket of HLA-DQ2 while the TG2 protein is degraded.

In addition to dietary gluten and HLA, the enzyme transglutaminase 2 (TG2) plays a central role in CeD pathogenesis by deamidating antigenic gluten peptides (Figure 1A), which dramatically enhances their affinity for HLA-DQ2/8. Blocking TG2 activity was shown to prevent gluten-induced tissue damage in the small intestine in a genetically engineered mouse model of CeD (1). More recently, an irreversible TG2 inhibitor, ZED-1227, was reported to prevent villus atrophy and crypt hyperplasia in patients with CeD challenged with daily gluten over a period of 6 weeks (2). While a compelling case can thus be made for a causative role of TG2 activity in CeD pathogenesis, the cellular sources of the active TG2 that generates deamidated gluten antigens in the celiac small intestine remains unclear. This question is complicated by the abundance of the TG2 protein in the small intestinal mucosa, both inside and outside cells (3). In the cytosol, TG2 is reversibly maintained in a catalytically inactive state by guanine nucleotide binding (4), whereas outside cells TG2 is inactivated by a different allosteric mechanism, a reversible disulfide bond (5). Recent studies have also implicated luminal TG2 as a possible source of pathogenically relevant activity in the celiac small intestine (6, 7).

We recently described a hitherto unrecognized mechanism, hereafter referred to as the TG2/low-density lipoprotein receptor-related protein 1 (LRP1) pathway, for gluten antigens to undergo simultaneous deamidation and lysosomal concentration in APCs (Figure 1B) (8). Interaction with low-density lipoprotein-related protein 1 (LRP1) has the remarkable effect of stabilizing a short-lived covalent intermediate between catalytically active TG2 and a suitable substrate for long enough to elicit canonical LRP1 receptor–mediated endocytosis while only allowing hydrolytic resolution of this covalent intermediate in the rapidly acidifying endosome. Guided by the hypothesis that the TG2/LRP1 pathway plays a major role in antigen presentation to HLA-DQ2 restricted inflammatory T cells in a patient with CeD, the goals of our present study were to: (a) identify cells with TG2/LRP1 pathway activity in the small intestine and the periphery; (b) determine spatial and temporal relationships between TG2 activity associated with the cellular TG2/LRP1 pathway and that which has been previously observed in the extracellular matrix (ECM) of the small intestine; and (c) demonstrate that at least a subset of these cells can capture dietary gluten antigens and present them to HLA-DQ2–restricted T cells (1, 9). A key finding of our study is that the dominant cells that harness the TG2/LRP1 pathway in the small intestine for gluten antigen presentation are identical to a population of dendritic cells (DCs) previously demonstrated to play a central role in the establishment of dietary antigen tolerance in mice (10, 11). They also show strong similarity to a DC population in the celiac small intestine that undergoes a marked increase in response to dietary gluten and can robustly activate gluten-specific T cells found exclusively in patients with CeD (12, 13).

## Results

### Oral HB-230 enables discrimination between ECM-associated and LRP1-associated TG2 activity in the mouse small intestine.

Prior to this report, virtually all investigations involving visualization of TG2 activity in the intact mammalian intestine utilized 5-biotinamidopentylamine (5-BP) as a probe (14). Because 5-BP is a cosubstrate for the TG2-catalyzed transamidation reaction, it biotinylates proteins with Gln (Q) residues for which TG2 has high substrate specificity. Furthermore, because extracellular TG2 in the small intestine is rapidly deactivated via disulfide bond formation (9), only ECM proteins in the vicinity of the site of TG2 secretion are labeled with 5-BP. Thus, in situ 5-BP labeling of intestinal mucosa serves as a convenient marker of TG2 activity, an inference that is underscored by strongly overlapping fluorescence signals from streptavidin and anti-TG2 antibodies in IHC analysis of intestinal tissues (1, 15).

**Figure 2:**
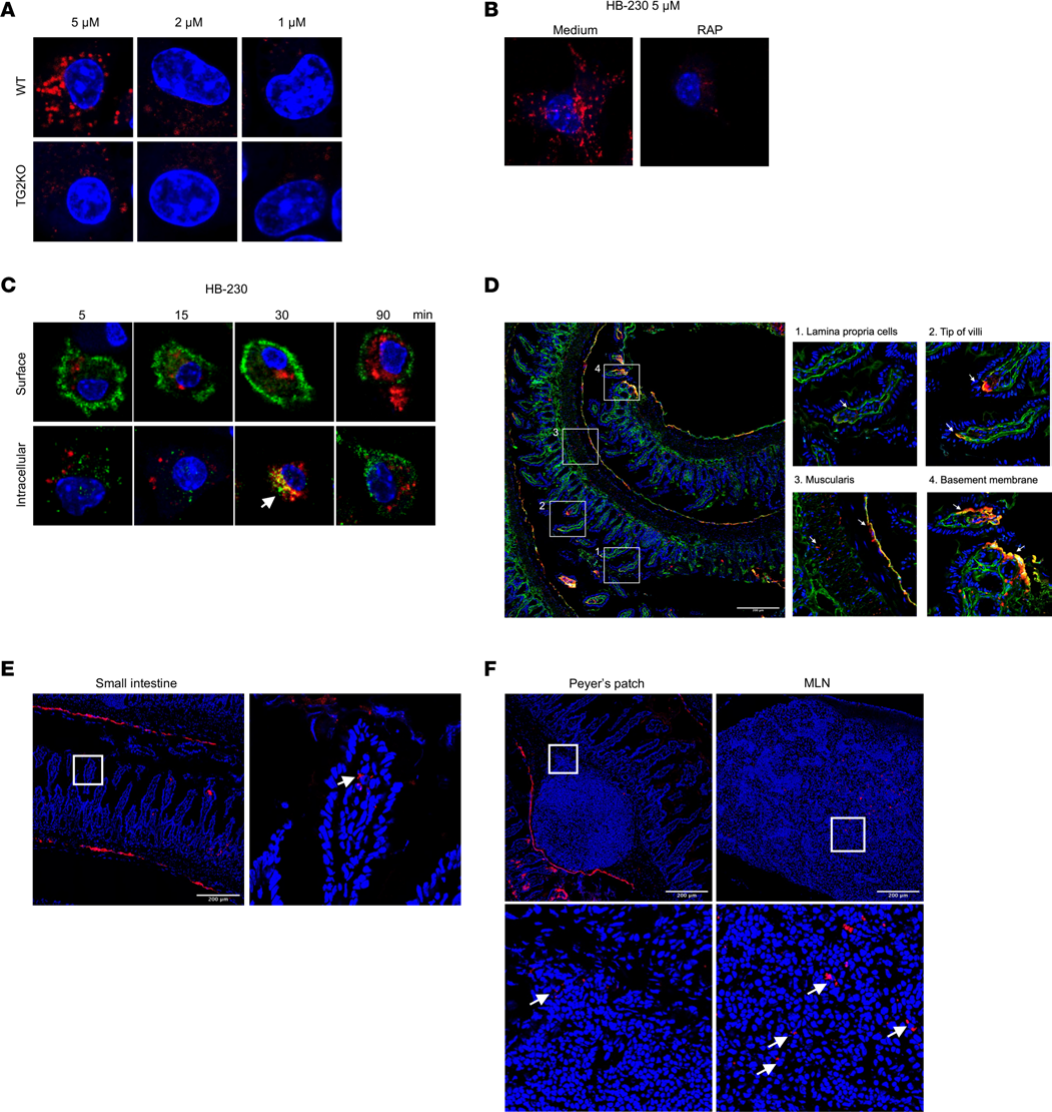
Detection of different active forms of TG2 by HB-230. (**A**) Bone marrow–derived dendritic cells (BMDCs) from WT and TG2 knockout (TG2-KO) mice were treated with increasing concentrations of HB-230 for 90 minutes. WT BMDCs showed prominent HB-230 puncta, corresponding to lysosomes, which were absent in TG2-KO cells. (**B**) WT BMDCs pretreated with the LRP1 antagonist RAP (5 μM of HB-230 and 3 μM of RAP, 30 minutes) exhibited reduced HB-230 puncta formation following 90 minutes of probe treatment, indicating LRP1 is required for lysosomal delivery of TG2. (**C**) WT BMDCs were incubated with HB-230 (2 μM) for 5–90 minutes and costained with anti-TG2 antibody (green) and DAPI (blue), with or without cell permeabilization to detect surface or intracellular TG2, respectively. Early time points showed low red/green signal at the surface, suggesting limited catalytic activity of TG2. In permeabilized cells, HB-230 and TG2 colocalized at intermediate time points, consistent with lysosomal processing of TG2. A ~90-minute movie of the entire uptake process by a representative BMDC is available as Supplemental Video 1. (**D**) WT mice were orally dosed with HB-230 (10 mg/kg), and tissues were collected 2 hours later. Ileal cryosections showed four HB-230 labeling patterns: (no. 1) lysosomal puncta in lamina propria, (no. 2) subepithelial extracellular matrix (ECM) colocalization with TG2 at villus tips, (no. 3) sporadic ECM labeling in the muscularis, and (no. 4) basement membrane of villi. (**E** and **F**) Additional HB-230–labeled cells were observed in the lamina propria (**E**) and Peyer’s patches and mesenteric lymph nodes (MLN) (**F**), with perinuclear puncta (white arrows) suggestive of lysosomal localization. All images were acquired at 40× magnification.

We recently engineered HB-230, a highly selective, irreversible inhibitor of catalytically active TG2 that also contains sulfocyanine 5 (sulfo-Cy5), a bright fluorophore with maximal absorption at 662 nm (8). The selectivity of HB-230 for TG2 is evident from the large, dose-dependent fluorescent puncta observed in bone marrow–derived DCs (BMDCs) from WT but not TG2-KO mice (Figure 2A). The requirement of LRP1 activity for puncta formation is underscored by their strong inhibition by receptor-associated protein (RAP), a selective inhibitor of LRP1-mediated endocytosis (Figure 2B). To visualize the source of catalytically active TG2 that recognizes HB-230, we pretreated WT BMDCs with HB-230 followed by costaining with an anti–mouse TG2 antibody. Under these conditions, puncta were observed to be initiated on the cell surface, followed by rapid endocytosis and postendocytic destruction of TG2, as would be expected for most proteins that are trafficked into the lysosome (Figure 2C). To provide a clearer view of the dynamics of HB-230 uptake by DCs, we performed time-lapsed microscopic analysis of HB-230 uptake by live BMDCs. A representative video (Supplemental Video 1; supplemental material available online with this article; https://doi.org/10.1172/jci.insight.196102DS1) shows the accumulation of puncta in endocytic vesicles within 10 minutes following exposure to the probe. Labeled endosomes merge with larger lysosomal compartments where the probe persists for up to 90 minutes. Three additional features of HB-230 enabling its oral efficacy as a probe of TG2 activity in the small intestine are its water solubility, acid stability and short systemic half-life (t_1/2_ ~ 10 minutes).

As a step toward studying the TG2/LRP1 pathway in the mouse small intestine, WT mice were given 10 mg/kg HB-230 orally after fasting overnight (~16 hours). Two hours after oral gavage of HB-230, the small intestine was removed and processed using Swiss-roll tissue preparation methods (Supplemental Figure 1A) prior to snap-freezing. From this Swiss-roll preparation, 5 μm cryosections were cut, costained with a polyclonal rabbit anti–mouse TG2 antibody, and imaged via confocal microscopy. Consistent with our prior observations with 5-BP, sporadic TG2 activity was detected in the ECM of the lamina propria (mostly near the villus tip) and the muscularis of the WT mouse small intestine (Figure 2D boxes 2-4) but not in TG2-KO mice (Supplemental Figure 1A). ECM-associated TG2 activity typically appears as a fluorescent yellow signal, presumably due to strong overlap between the red (HB-230) and green (anti-TG2 antibody) fluorophores. Notably, WT (but not TG2-KO) mice also contained few sporadic cells with strong HB-230 labeling in the lamina propria (LP) (Figure 2D). Unlike ECM-associated TG2 activity, this form of HB-230 labeling does not overlap with IHC staining of the TG2 protein, suggesting that the corresponding cells may be capable of lysosomal degradation of labeled TG2 in an LRP1-dependent manner.

To better understand the dynamics of tissue distribution and localization of HB-230 staining following its oral administration, draining Peyer’s patches and mesenteric lymph nodes (MLNs) were examined from WT and TG2-KO mice dosed orally with HB-230. The small intestine containing Peyer’s patches was dissected 2 hours after dose and processed using Swiss-roll tissue preparation before being snap-frozen. MLNs were collected, embedded, and snap-frozen separately. The Swiss-rolled small intestine provided a detailed view of the tissue with the most structure, showcasing cross-sections of villi and Peyer’s patches. The ileum was centered, with the spiral leading outward into the distal jejunum (Supplemental Figure 1A). HB-230 puncta (indicative of LRP1-associated TG2 activity) were sporadically distributed throughout the mouse small intestine, from the jejunum to the ileum, with relatively few occurrences in the distal jejunum (Figure 2E and Supplemental Figure 1A). No punctate HB-230 staining was observed in TG2-KO tissue, indicating selective labeling of active TG2 by HB-230 (Supplemental Figure 1A). After 2 hours of HB-230 administration, small numbers of punctate and HB-230–labeled cells were observed in the villi (Figure 2E), as were Peyer’s patches and MLNs (Figure 2F) from WT mice. By 24 hours, the presence of labeled cells was limited to the MLN (Supplemental Figure 1B). These observations suggest that either the endocytosed fluorophore had been metabolized or otherwise eliminated, or that the labeled cells — potentially migratory DCs — had migrated out of the lamina propria and Peyer’s patches after internalizing the TG2–HB-230 complex.

### CD103^+^ DCs are the predominant source of LRP1-associated TG2 activity in the mouse small intestine.

**Figure 3:**
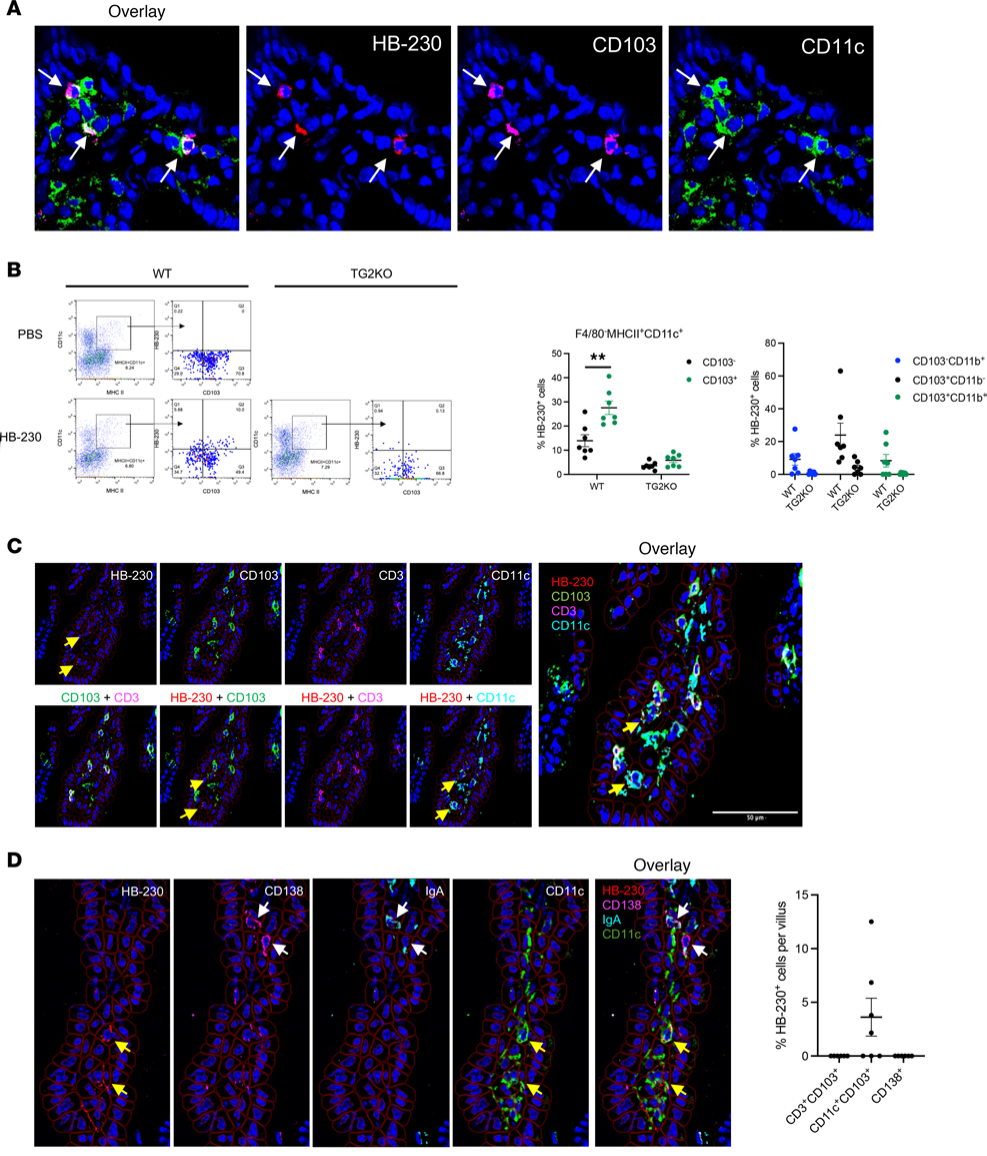
CD103^+^ DCs are predominantly labeled by HB-230 in intestinal lamina propria. Mice received HB-230 at a dose of 10 mg/kg via oral administration. Two hours after administration, small intestines were collected for either snap freezing or the isolation of CD45^+^ cells from the lamina propria. (**A**) WT ileum cryosections were costained with anti-CD103 and anti-CD11c antibodies. White arrows indicate punctate HB-230–labeled cells costained with DC markers. (**B**) Viable CD45^+^ immune cells were enriched from the small intestines of HB-230–dosed WT and TG2-KO mice. DCs were gated by MHCII^+^CD11c^+^F4/80^–^ cells, and the percentage of HB-230 among CD103^+^ cells with or without CD11b was analyzed based on PBS control (WT, *n* = 7; TG2-KO, *n* = 7). Small intestines from HB-230–administered WT mice were costained with various antibodies (Table 1) using the PhenoCycler multiplex staining system for immune cell characterization. (**C**) To verify that HB-230^+^CD103^+^ cells were DCs and not T lymphocytes, tissue sections were additionally stained with an anti-CD3 antibody. Yellow arrows show that HB-230 puncta colocalizes with CD103^+^CD11c^+^ DCs but not with CD3^+^CD103^+^ lymphocytes. (**D**) To verify that plasma cells did not take up HB-230 to form puncta, tissue sections were additionally stained with an anti-CD138 antibody. Yellow arrows denote HB-230 puncta colocalized with CD11c^+^ DCs but not with CD138^+^IgA^+^ plasma cells (indicated by white arrows). The dot plot shows the percentage of HB-230 among the cell population of each villus. *P* values were determined using an unpaired Mann-Whitney 2-tailed *t* test. **P < 0.01. Data represent mean ± SEM. **A** was captured under a confocal microscope at 40× magnification, and **C** and **D** were captured at 20× magnification.

Migratory CD103^+^ DCs are known for sampling dietary antigens in the lamina propria of the small intestine and migrating to draining MLNs, where they activate dietary-specific T cells, particularly driving Th1 type immunity to dietary antigens, including gluten, in the presence of inflammatory triggers (10, 11, 16–19). To determine whether CD103^+^ DCs efficiently uptake HB-230 in vivo, we costained tissue sections with anti-CD103 and anti-CD11c antibodies. Confocal microscopy clearly demonstrated that HB-230^+^ cells were predominantly labeled with an anti-CD103 antibody, and these CD103^+^ cells were costained with CD11c (Figure 3A). To verify this finding, we isolated lamina propria cells from WT and TG2-KO mice dosed orally with HB-230 and subjected them to flow cytometric analysis. After removing dead cells and debris, CD45^+^ cells were enriched using magnetic beads. No significant differences in the percentage of DCs and macrophages were observed in WT and TG2-KO mice (Supplemental Figure 2A). Focusing on the CD11c^+^MHCII^+^F4/80^–^ population, HB-230 was observed to be taken up most effectively by the CD103^+^ subpopulation of DCs in WT mice (Figure 3B). An insignificant fraction of equivalently labeled cells was detected in TG2-KO mice (Figure 3B). In contrast to CD103^+^ DCs, MHCII^+^F4/80^+^ macrophages in the small intestine also showed measurable uptake of HB-230, but the difference in labeling frequency between macrophages from WT and TG2-KO mice was not significant (Supplemental Figure 2B). Intestinal CD11c^+^MHCII^+^CD103^+^ DCs can be subclassified into 2 types of classical or conventional DCs (cDCs): cDC1 (CD11b^–^) and cDC2 (CD11b^+^) (20, 21). Among F4/80^–^CD11c^+^MHCII^+^ DCs, 24% of cDC1 are HB-230^+^, and 8% of cDC2 are HB-230^+^ (Figure 3B).

To assess whether the TG2/LRP1 pathway was active in other types of cells in the lamina propria, we utilized multiplex IHC staining of tissues from HB-230–dosed mice. Our 22-plex panel includes lymphocytes (plasma cells, B cells, and T cells) in addition to other relevant cell types (epithelial cells, endothelial cells, lymphatic endothelial cells, and fibroblasts) as well as extracellular matrix components (TG2 protein, collagen 1A1).

Intraepithelial lymphocytes have been found to express CD103 (αEβ7 integrin) to mediate T cell adhesion to epithelial cells through its binding to E-cadherin. To investigate whether HB-230^+^ puncta appeared in CD103^+^ T lymphocytes, we examined the CD3^+^CD103^+^ population. None of the CD3^+^CD103^+^ T cells were found to have HB-230 puncta (Figure 3C).

Because TG2-specific plasma cells have also been observed in substantial numbers in small intestinal biopsies of patients with CeD (22, 23), we also sought to test whether the plasma cells present in our samples had an active TG2/LRP1 pathway in the absence of inflammation. For this, tissue samples from WT mice were costained with CD138 and IgA to identify IgA-producing plasma cells within the lamina propria. No HB-230 labeling was detected in CD138^+^IgA^+^ plasma cells (Figure 3D).

### ECM-associated TG2 activity colocalizes with Type I collagen in lamina propria.

Upon secretion by *TGM2*-expressing cells, TG2 interacts with extracellular matrix proteins, including fibronectin and collagens (24). Type I collagen is a major ECM protein in the small intestine, where it supports epithelial cell growth (25). To identify possible sources of the ECM-associated TG2 activity shown in Figure 2D, frozen tissue sections of the small intestine from HB-230–dosed WT mice were first costained with antibodies against TG2 and Type I collagen. As expected, in the lamina propria of these sections (Supplemental Figure 3A) a clear overlap was observed between sporadic HB-230 (red) and TG2 protein (green); this form of active TG2 also colocalized with Type I collagen. Analogous to ECM-associated HB-230, HB-230 colocalized with TG2 and Type I collagen was observed along the outer layer of muscularis in the small intestine (Supplemental Figure 3).

Because fibroblasts are preferentially localized on the sub-epithelial side of the basement membrane in small intestinal villi (26), we costained HB-230–dosed small intestinal tissue sections with an antibody against platelet-derived growth factor receptor α (PDGFRα), a well-established fibroblast marker. Indeed, the sporadic HB-230 observed along the basement membrane frequently colocalized with PDGFRα^+^ cells (Supplemental Figure 3B).

Because the *TGM2* gene is expressed by many cells in the small intestine, we sought to inquire whether other cell types might also contribute to the TG2 activity revealed by HB-230. Antibodies against E-cadherin, α-smooth muscle actin (αSMA), lymphatic vessel endothelial hyaluronan receptor-1 (LYVE-1), and CD31 were used to identify epithelial cells, smooth muscle cells or fibroblasts, lymphatic endothelial cells, and blood vessel endothelial cells, respectively. Whereas a very small percentage of the abundant epithelial and endothelial cells showed HB-230 positivity, some αSMA^+^ cells were colocalized with HB-230 (Supplemental Figure 3C). These αSMA^+^ cells were observed within the villi, crypts, and muscularis and are presumably of a fibroblast origin.

### Gluten antigens are presented to T cells in MLNs by CD103^+^ DCs.

To investigate whether lamina propria-derived CD103^+^ DCs with an active TG2/LRP1 pathway are capable of gluten antigen presentation in mesenteric lymph nodes, we used a humanized DR3.DQ2 mouse that harbors the human HLA-DQ2.5 genes while lacking endogenous mouse MHCII genes (27). A cohort of these transgenic mice was maintained initially under gluten-free conditions, followed by a 14-day enhanced gluten diet in which a well-characterized 33-mer gluten peptide harboring multiple HLA-DQ2.5 epitopes (LQLQPFPQPQLPYPQPQLPYPQPQLPYPQPQPF; ref. 28) was dosed by oral gavage every other day. On the final day of the study, mice were also dosed with 10 mg/kg HB-298, a sulfo-Cy5–conjugated analog of this 33-mer peptide, either 2 hours or 24 hours prior to sacrifice. Their small intestines, Peyer’s patches, and MLNs were dissected and snap-frozen for imaging.

**Figure 4:**
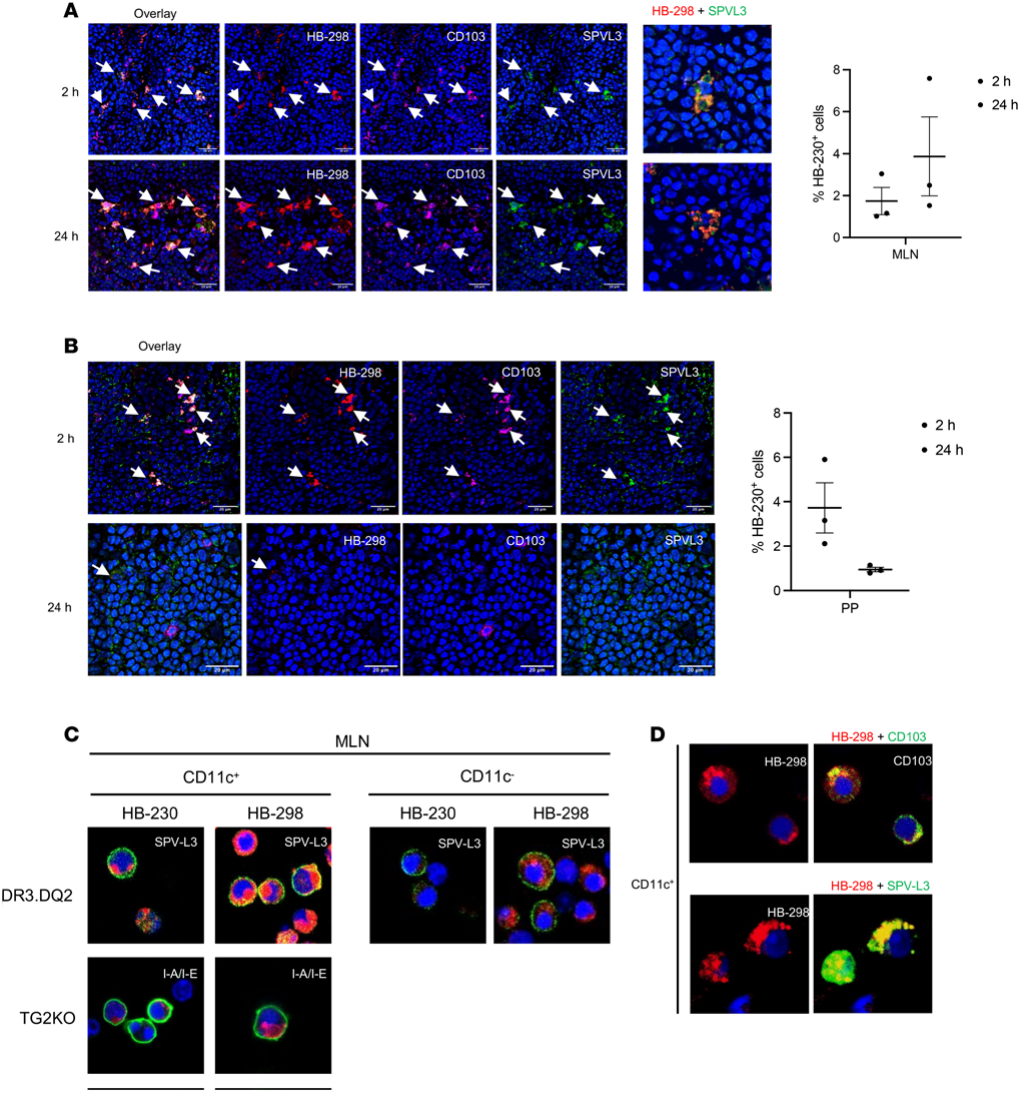
CD103^+^ DCs present gluten peptides in MLN in an HLA-DQ2–restricted manner. DR3.DQ2 mice were orally dosed with 10 mg/kg of HB-298 (a fluorescent analog of an immunodominant 33 mer gluten peptide harboring multiple epitopes recognized by HLA-DQ2) either 2 or 24 hours prior to sacrifice. After euthanasia, mesenteric lymph nodes (MLNs) and Peyer’s patches (PP) were collected, snap-frozen, and stained with antibodies. (**A** and **B**) MLN and PP from DR3.DQ2 mice following HB-298 administration were costained with antibodies targeting CD103 (magenta) and HLA-DQ2 (SPV-L3, green). White arrows indicate HB-298 labeling of DQ2^+^CD103^+^ DCs. A focal image showed HB-298 colocalized with HLA-DQ2 (shown in yellow) (**A**). Quantification of labeled cells from MLN and PP confirmed that, whereas the prevalence of HB-298–labeled cells remain unchanged in the MLN at the 24-hour time point, analogously labeled cells in the PP had migrated out of this compartment by 24 hours. (**C** and **D**) CD11c^+^ and CD11c^–^ cells isolated from the MLNs of DR3.DQ2 and TG2-KO mice were incubated with 2 μM of HB-230 or HB-298 for 90 minutes. (**C**) Thereafter, MLN cells from DR3.DQ2 mice were stained with antibodies against HLA-DQ2 (SPV-L3, green), while cells from TG2-KO mice were stained with antibodies against mouse MHCII (I-A/I-E, green). Cell-surface gluten peptide presentation (indicated by a yellow cell boundary) was noted in HB-298–treated CD11c^+^ cells from DR3.DQ2 mice but not in HB-230–treated cells or in TG2-KO mice. (**D**) HB-298–treated MLN cells from DR3.DQ2 mice were also stained with anti-CD103 (green, upper panel) or anti-DQ2 (SPV-L3, green, lower panel) antibodies in CD11c^+^ cells from MLNs of DR3.DQ2 mice. Here, too, strong antigen presentation by CD103^+^ DCs was noted. Representative images were captured using a confocal microscope at 40× magnification. Data represent mean ± SEM.

In contrast to HB-230 uptake by CD103^+^ DCs in the lamina propria, very few HB-298^+^ cells were detected in the small intestinal villi of this cohort of mice (data not shown). Remarkably though, a significant number of HB-298^+^CD103^+^ cells were observed in the MLNs, and their abundance remained relatively unchanged between 2 hours and 24 hours (Figure 4A). A few double-positive cells were also observed in Peyer’s patches 2 hours after dosing, but their numbers declined by 24 hours (Figure 4B). To establish whether HB-298 persisted in the MLN by virtue of its ability to bind to HLA-DQ2, we costained tissues with the SPV-L3 antibody that specifically recognizes HLA-DQ2. This antibody strongly colocalized with both the anti-CD103 antibody and HB-298 in the MLNs and Peyer’s patches (Figure 4, A and B). A similar control experiment performed with WT mice revealed far fewer HB-298^+^ cells in the MLN (Supplemental Figure 4A). Our data suggest that HLA-DQ2^+^CD103^+^ DCs are exquisitely well equipped to sample dietary gluten antigens in the small intestine and/or Peyer’s patches and to later migrate to the MLNs to enable antigen presentation.

To verify the extraordinary capacity of these DCs to present dietary gluten antigens, we isolated CD11c^+^ DCs along with CD11c^–^ cells as controls from the MLNs of both DR3.DQ2 and TG2-KO mice. These cells were incubated ex vivo with either 2 μM HB-230 or HB-298 for 90 minutes, followed by staining with antibodies against their cognate MHCII molecules. Only HLA-DQ2–expressing CD11c^+^ cells were able to present the fluorescent gluten peptide (but not HB-230) on their cell surface (Figure 4C). Neither CD11c^–^ cells from DR3.DQ2 mice nor CD11c^+^ cells from TG2-KO mice showed significant colocalization of HB-298 with their cell-surface MHCII, although peptide uptake was observed in some of these cells. As expected, HB-230 uptake was higher in CD11c^+^ cells from DR3.DQ2 mice than in either CD11c^–^ cells from DR3.DQ2 mice or CD11c^+^ cells from TG2KO mice, although the fluorophore was not present on the surface of CD11c^+^HLA-DQ2^+^ cells. The CD103 positivity of these double-positive DCs was also confirmed (Figure 4D).

### Dietary gluten upregulates ECM-associated and DC-associated TG2 activity in the small intestine of HLA-DQ2 mice.

Certain viral infections and other inflammatory triggers have been shown to increase TG2 activity in the small intestine (1, 18). However, the relationship between HLA-DQ2, gluten and TG2, the 3 principal pathogenically relevant molecules in CeD, is unknown. We therefore sought to test the hypothesis that dietary gluten alone can activate either the cell-associated TG2/LRP1 pathway or ECM-associated TG2 activity (or both) in an HLA-DQ2.5 background. Although the T cell response to dietary gluten in DQ2 mice is expected to be tolerogenic in nature, it enables testing whether HLA-DQ2 and TG2 activities can be mutually reinforcing in the presence of dietary gluten.

Parental WT and DR3.DQ2 mice were maintained on a GFD prior to weaning the pups, and all offspring were maintained on a GFD until initiation of the study. Our 14-day study involved 3 cohorts of mice for each genotype — a GFD and an enhanced gluten diet (described above). After 14 days, an oral gavage of 10 mg/kg of HB-230 was given 2 hours prior to sacrifice and harvesting of the small intestines.

**Figure 5:**
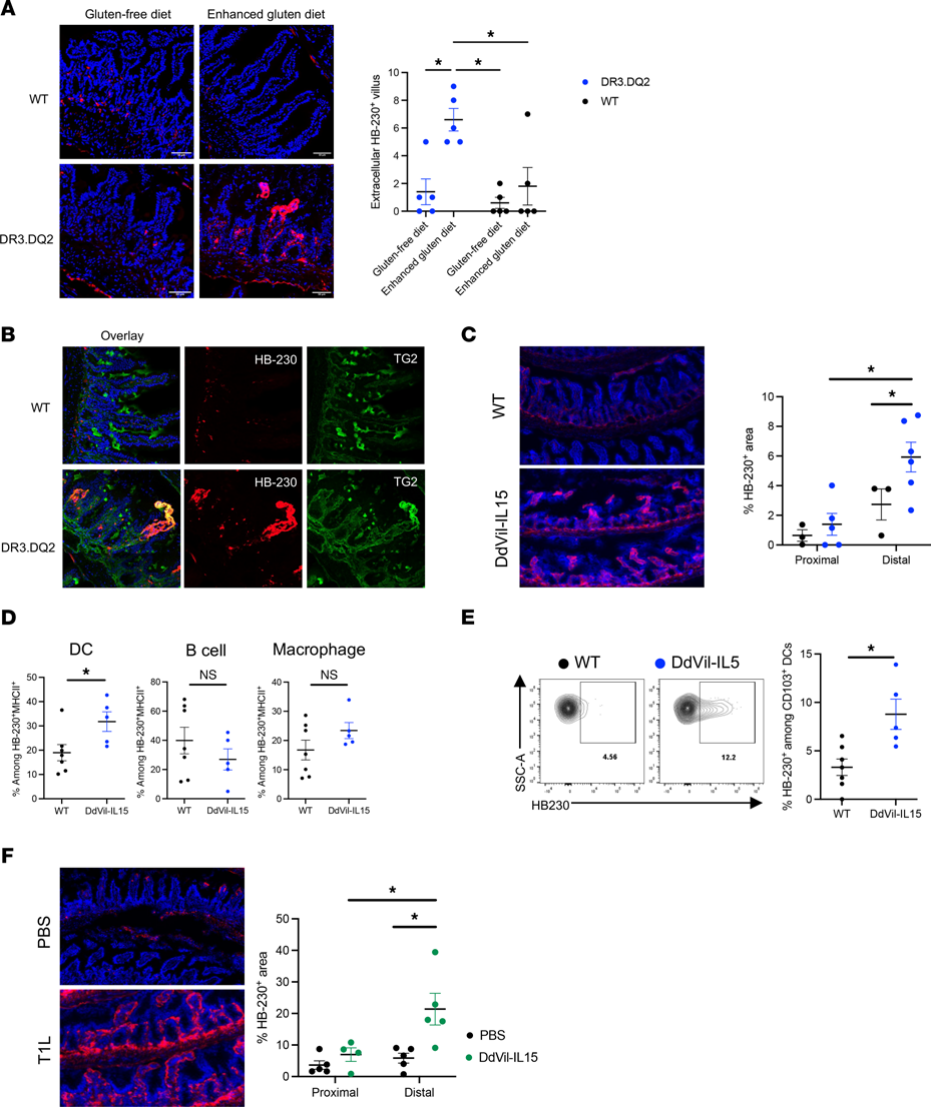
Enhanced gluten diet, reovirus infection, and IL-15 overexpression increase TG2 activity in the small intestine. (**A** and **B**) WT and DR3.DQ2 mice were fed control (gluten-free) or gluten-enriched diets for 14 days prior to oral HB-230 administration (10 mg/kg). Two hours later, tissues were collected and stained. (**A**) DR3.DQ2 mice on a gluten-enhanced diet showed increased HB-230 labeling at villus tips. Dot plot quantifies extracellular HB-230^+^ villi per 10 villi. (**B**) Confocal imaging revealed strong colocalization of HB-230 with TG2 at villus tips, indicating increased ECM-associated TG2 activity. *P* values were determined using a 2-way ANOVA, and multiple comparisons were assessed using Benjamini, Krieger, and Yekutieli’s 2-stage step-up method. *P < 0.05. Data represent mean ± SEM. (**C**–**E**) DdVil-IL15 transgenic and WT mice received HB-230 (10 mg/kg), and intestines were collected 2 hours later. (**C**) Quantification of HB-230^+^ signal (pixel area relative to total tissue area) in proximal and distal intestine sections revealed increased TG2 activity in DdVil-IL15 mice. (**D**) Flow cytometry of lamina propria cells showed a higher frequency of CD11c^+^ DCs, but not CD19^+^ B cells or F4/80^+^ macrophages, among total HB-230^+^CD45^+^MHCII^+^ cells in DdVil-IL15 mice. (**E**) Representative flow cytometry plots of HB-230^+^CD103^+^ DCs in WT (*n* = 8) vs. DdVil-IL15 (*n* = 5) mice. *P* values were determined using a 2-way ANOVA, and multiple comparisons were assessed using Benjamini, Krieger, and Yekutieli’s 2-stage step-up method. *P < 0.05. Data shown as mean ± SEM. (**F**) WT mice were infected with T1L reovirus or treated with PBS 46 hours prior to HB-230 administration. Confocal images showed increased extracellular HB-230 staining in distal intestines of reovirus-infected WT mice. *P* values were determined using a 2-way ANOVA, and multiple comparisons were assessed using Benjamini, Krieger, and Yekutieli’s 2-stage step-up method. *P < 0.05. Data shown as mean ± SEM.

No significant differences were observed in small intestinal TG2 activity between WT and DR3.DQ2 mice that were fed a GFD. In contrast, the enhanced gluten diet led to a marked increase in ECM-associated TG2 activity in DR3.DQ2 but not WT mice (Figure 5A and Supplemental Figure 5A). Gluten-dependent upregulation of TG2 activity was most pronounced in the ECM (Figure 5B), although the prevalence of punctate HB-230^+^ cells in the lamina propria also increased (Supplemental Figure 5B).

### Reovirus infection or IL-15 overexpression also upregulates TG2 activity in the small intestine.

IL-15 is an inflammatory cytokine upregulated in reovirus infection; it also plays a critical role in CeD pathogenesis (1, 29). Transgenic DdVil-IL15 mice overexpress IL-15 driven by the MHC class I promoter Dd and the intestinal epithelium-specific villin promoter, which specifically increases IL-15 expression in their small intestinal epithelial cells and lamina propria APCs. Again, although DdVil-IL15 mice do not reflect all the immunologic features of CeD, they facilitate analysis of the relationship between TG2 activity and a disease-relevant cytokine. By quantifying HB-230^+^ areas in the small intestine tissue, it was determined that IL-15 overexpression enhanced TG2 activation, particularly in the distal small intestine (Figure 5C and Supplemental Figure 6A).

To analyze the effect of IL-15 overexpression on the prevalence of professional APCs and the activity of their TG2/LRP1 pathway, we also performed flow cytometric analysis on isolated lamina propria cells from additional cohorts of DdVil-IL15 and WT mice dosed with HB-230 prior to sacrifice. DdVil-IL15 mice had more DCs among HB-230^+^MHCII^+^ cells in their lamina propria compared with their WT counterparts, whereas no differences were observed in the prevalence of macrophages or B cells between the 2 strains (Figure 5D). In particular, an enrichment of HB-230^+^ migratory CD103^+^ DCs among CD11c^+^MHCII^+^ DCs was observed in response to IL-15 overexpression (Figure 5E). Among these CD103^+^ DCs, CD103^+^CD11b^+^ cDC2 showed a larger increase in HB-230 positivity (Supplemental Figure 6B) than CD103^+^XCR1^+^ cDC1 (Supplemental Figure 6C).

Reovirus infection has been shown to activate TG2 in the small intestine, as assessed with the 5-BP probe (introduced above) (18). To confirm this, type1 Lang (T1L) reovirus–infected mice and mock-infected controls were dosed orally with HB-230 followed by histologic analysis of Swiss-roll sections (Supplemental Figure 7A). A significant increase in ECM-associated HB-230 was observed in tissue derived from reovirus-infected mice (Figure 5F). TG2 activity was predominantly localized to the distal small intestine, particularly in the ileum and distal jejunum (Supplemental Figure 7B). Surprisingly, T1L-infected WT and TG2-KO mice had more HB-230^+^ cells among macrophages but not in DCs (Supplemental Figure 7, C and D). In addition, CD103^+^CD11b^+^ cDC2 also showed a larger increase in TG2/LRP1 activity than CD103^+^XCR1^+^ cDC1 (Supplemental Figure 7, E and F).

### HB-230 detects activity of the TG2/LRP1 pathway in human peripheral blood cells.

The above studies demonstrate that a subset of DCs in the small intestinal mucosa of mice are especially well suited for sampling dietary gluten antigens by virtue of their elevated TG2/LRP1 pathway activity. An earlier analysis of duodenal biopsies from gluten-challenged patients with CeD showed an increased number of CD14^+^CD11c^+^ DCs in the lamina propria (12). We therefore sought to assess whether CD14^+^CD11c^+^ DCs in human peripheral blood mononuclear cells (PBMCs) had an active TG2/LRP1 pathway. A total of 4 patients with CeD on a GFD and 3 healthy volunteers were included in this study. PBMCs were incubated with 2 μM HB-230 or PBS for 90 minutes. After 2 washes, the cells were stained with an appropriate antibody cocktail and analyzed via flow cytometry. HB-230 positivity was regarded as a marker of cellular TG2/LRP1 pathway activity.

**Figure 6:**
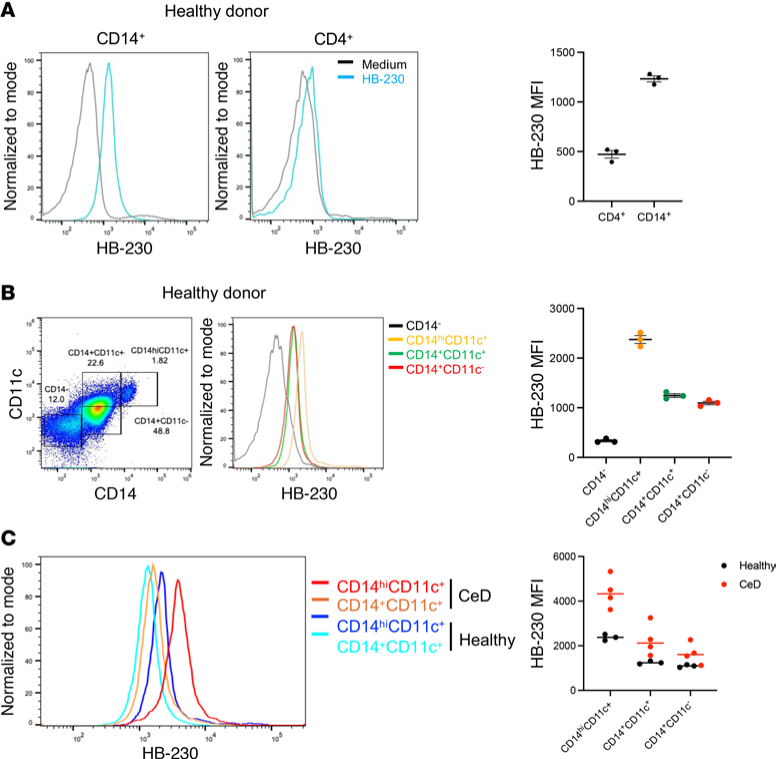
HB-230 uptake by peripheral CD14^+^ monocytes is increased in patients with celiac disease compared with healthy controls. Whole blood from either CeD or healthy patients was collected. Red blood cells were removed, and the remaining cells were incubated with HB-230 for 90 minutes. Following incubation, cells were stained with antibodies and analyzed using a flow cytometer. (**A**) The histogram displays the fluorescence intensity of HB-230, and the dot plot shows the mean fluorescence intensity (MFI), among CD4^+^ T cells or CD14^+^ monocytes from healthy donors. (**B**) The dot plot shows the monocyte gating, and the histogram shows the MFI of HB-230 among CD14^–^ cells (black), CD14^hi^CD11c^+^ (yellow), CD14^+^CD11c^+^ monocytes (green), and CD14^+^CD11c^–^ monocytes (red) from healthy donors. The dot plot displays the MFI of HB-230 among different populations. (**C**) The histogram displays the fluorescence intensity of HB-230 among CD14^hi^CD11c^+^ (red from the patient with CeD and blue from the healthy control) or CD14^+^CD11c^+^ monocytes (orange from the patient with CeD and cyan from the healthy control). The dot plot displays the MFI of HB-230 among different populations from patients with CeD (*n* = 4) or healthy controls (*n* = 3). Data represent mean ± SEM.

In contrast to CD4^+^ T lymphocytes, for example, which lacked detectable TG2/LRP1 pathway activity, a majority of CD14^+^ cells from both nonceliac controls and patients with CeD accumulated HB-230 (Figure 6A). Both CD11c^+^ cells (DCs) and CD11c^–^ cells (monocytes, macrophages) within this population of CD14^+^ cells showed comparable levels of TG2/LRP1 pathway activity (Figure 6B). In healthy subjects as well as patients with CeD, a subpopulation of CD14^+^ cells had high levels of this marker (CD14^hi^); the TG2/LRP1 pathway was correspondingly more active in these cells (Figure 6, B and C). Thus, cells of the monocytic lineage in human peripheral blood appear to have an active TG2/LRP1 pathway.

## Discussion

For many years now, the central role of the inflammatory T cell response to dietary gluten antigens in CeD has been widely recognized (30, 31). Two proteins are known to play an essential role in this immune recognition process — HLA-DQ2.5 (or, less frequently, HLA-DQ8), and the enzyme TG2. Whereas the molecular basis for their association with CeD is well understood (3, 32), their pathogenically relevant cellular origins have remained a mystery. Our findings reported here provide insights into both these questions.

HB-230 is a recently reported small molecule fluorophore that is also a highly selective, active site-directed, irreversible inhibitor of TG2 (8). By developing protocols to use it as a probe of catalytically active TG2 in the small intestine of mice, we have uncovered 2 principal sources of mucosal TG2 activity. One source of active TG2 is ECM associated and colocalizes with collagen deposits in the basement membranes that line individual villi; it is likely secreted by fibroblasts that play a key role in fabricating this subepithelial structure (26). The other pool of TG2 targeted by HB-230 is localized to CD103^+^ DCs in the lamina propria; its punctate intracellular appearance stems from receptor-mediated endocytosis and lysosomal delivery via the action of the TG2/LRP1 pathway (Figure 1). Both sources of TG2 activity are sporadic in a healthy small intestine, comprising a small fraction of the total TG2 protein in this organ.

The ability of active TG2 on the surface of DCs to promote LRP1-mediated endocytosis of gluten antigens represents a highly effective mechanism for gluten antigen presentation to CD4^+^ T cells because it simultaneously catalyzes peptide deamidation while also concentrating the antigen in the lysosomal compartment of this important class of APCs. Among different subpopulations of intestinal DCs, CD103^+^ DCs are known to be especially important in the recognition of dietary antigens. Indeed, we have previously reported that they represent a major class of APCs that recognize gluten-derived antigens in mice with an appropriate MHC background (27). Using HB-298, a fluorescent 33-residue gluten peptide harboring multiple immunodominant T cell epitopes, we demonstrate that these DCs not only are highly capable of gluten antigen uptake and presentation on HLA-DQ2.5, but that they also migrate to mesenteric lymph nodes where they presumably instruct T cells to assume a tolerogenic or inflammatory phenotype.

Although TG2 is an abundant protein in the ECM of lamina propria in healthy mice, it is predominantly maintained in an inactive state (33). A variety of proinflammatory triggers are known to upregulate ECM-associated TG2 activity in lamina propria (1, 9, 18). In active CeD, this pool of TG2 can therefore be expected to contribute significantly to the production of deamidated gluten peptides in the subepithelial compartment, thereby enabling antigen presentation by other immune cells (e.g., B cells) (34).

Our findings also demonstrated that both epithelial overexpression of IL-15 and reovirus infection markedly increased HB-230 labeling in the distal small intestine, indicative of ECM-associated TG2 activation. Interestingly, while IL-15 overexpression also enhanced TG2 uptake in migratory DCs and macrophages, reovirus infection, despite robust ECM-associated TG2 activity, did not significantly enhance TG2 internalization in either cell subset. These findings suggest that, while ECM-associated TG2 activity can be induced by multiple inflammatory stimuli, only specific environmental contexts promote concurrent activation of the TG2/LRP1 pathway in APCs.

Notably, our findings described in this report prompt reconsideration of HLA-DQ2 mice as models for aspects of CeD. While several laboratories have reported the construction and characterization of this strain of transgenic mice, their inability to mount an immune response to dietary gluten has limited their use in CeD research. Using molecularly defined oral antigens at carefully titrated doses, we show that HLA-DQ2 mice can serve as a good model for the loss of oral tolerance to dietary gluten that is observed in patients with CeD.

Last but not least, our analysis of DCs found in peripheral blood of humans has confirmed that some circulating CD14^+^CD11c^+^ monocytes have an active TG2/LRP1 pathway, presumably allowing them to capture antigens harboring TG2 recognition motifs. Activity of this pathway appears to correlate with CD14 expression levels, suggesting that immature DCs are particularly dependent on this pathway for sampling circulating antigens. This finding speaks to the evolutionary relevance of TG2, an enigmatic protein whose biological role remains uncertain due to the lack of phenotypes associated with TG2-KO mice (15). It also presents an opportunity to identify and study circulating DCs that originate from the celiac intestine.

The findings reported here prompt several questions. For example, what is the nature of the catalytically active TG2 localized on the surface of DCs? How does it enable potent activation of endocytosis by the LRP1 receptor in an antigen-dependent manner? Do CD103^+^ migratory DCs in the small intestine transform into inflammatory DCs in response to signals that lead to a breakdown of oral tolerance in patients with CeD, or do inflammatory DCs in patients come from the periphery (12, 13)? How do CD103^+^ DCs upregulate ECM-associated TG2 activity? The tools and approaches described in this study represent powerful starting points for future studies aimed at addressing these questions.

## Methods

### Sex as a biological variable.

For human and mouse studies, both male and female patients were included.

### Animals.

Age and sex-matched (8- to 12-week-old) C57BL/6J WT mice were from The Jackson Laboratory (JAX000664) and maintained by in-house breeding. DR3.DQ2 (27) and TG2-KO (35) mice were housed in a specific pathogen-free environment at Stanford University under a protocol approved by the Administrative Panel on Laboratory Animal Care (APLAC). DdVil-IL15 transgenic mice (1) were maintained under specific pathogen-free conditions at the University of Chicago.

### Reagents.

HB-230 and HB-298 (LQLQPFPQPQLPYPQPQLPYPQPQLPYPQPQPF with the sulfo-Cy5 dye conjugated to the N-terminus) were synthesized using general peptide synthesis protocols previously described (8). HB-230 was dissolved in PBS as a 10 mg/mL solution for oral administration. HB-298 was dissolved in water as a 1 mg/mL solution for oral administration. The intact 33-mer gluten peptide cited above was also used as a supplement in an enhanced gluten diet, where it was dissolved in water as a 1 mg/mL solution and administered by oral gavage every other day.

### BMDC differentiation, stimulation, and analysis.

Bone marrow cells were collected from the femurs and tibias of mice using a 26 G needle attached to a 1 mL syringe filled with PBS. The red blood cells were removed using a red blood cell lysis buffer (BioLegend), and the remaining cells were resuspended in RPMI 1640 medium containing 10% fetal bovine serum and 1% antibiotics. These cells were plated in a petri dish at a concentration of 2 million cells per 10 mL of medium and supplemented with 10 ng/mL of GM-CSF for 8 days. On days 3 and 6, 10 mL of fresh GM-CSF-containing medium was added, and the suspension cells were harvested on day 7. Two hundred thousand BMDCs were plated in a 0.01% poly-L-lysine precoated 24-well glass-bottom dish and incubated with 2 μM HB-230 for 90 minutes. The cells were rinsed with PBS twice, stained with antibodies, and then fixed with 2% paraformaldehyde for 10 minutes, followed by staining with 4’,6-diamidino-2-phenylindole (DAPI). Stained cells were visualized and analyzed using a Zeiss LSM 980 confocal microscope (Zeiss) and Fiji ImageJ software.

### Live cell imaging.

Two hundred thousand BMDCs were plated in a poly-L-lysine precoated 24-well glass-bottom dish. The cells were then loaded in a Zeiss LSM 780 confocal microscope fitted with a chamber suitable for live cell imaging, maintained at 5% CO_2_ and 37°C. An initial image of a representative area of cells was taken. Then, 2 μM HB-230 was added and imaging of the cells was immediately initiated. Cells were imaged continuously at 1-minute intervals over 90 minutes. Live cell imaging raw data was processed using Fiji ImageJ software.

### Immunocytochemistry.

BMDCs were plated on a 0.01% poly-L-lysine precoated glass-bottom culture dish and incubated with 2 μM HB-230 for 5, 15, 30, and 90 minutes. After incubation, the medium was aspirated, and BMDCs were rinsed with PBS before surface TG2 staining. Paraformaldehyde-fixed and Triton X-100-permeabilized BMDCs were incubated with 2% fetal bovine serum to block nonspecific antigens prior to staining intracellular TG2 proteins. Colocalization of HB-230 and TG2 proteins was visualized and analyzed using a Zeiss LSM 980 confocal microscope and Fiji ImageJ software.

### Tissue collection and Swiss-rolled small intestine preparation.

Mice were fasted overnight for 14-16 h before receiving oral gavage dosing. Each mouse received a single bolus dose of HB-230 or HB-298 at 10 mg/kg via oral gavage either 2 h or 24 h before sacrifice. After the oral dosing, the mice were euthanized using carbon dioxide and dissected. The entire small intestine was removed, with fat and mesenteric lymph nodes discarded. The small intestine was cut into 2 segments. Segment 1 included the duodenum to the distal jejunum, while Segment 2 encompassed the remainder of the jejunum to the ileum. Each segment was cut open lengthwise and flushed with PBS to eliminate luminal residue. The distal jejunum from Segment 1 and the ileum from Segment 2 were positioned centrally and spiraled up. The rolled tissue was embedded in the OCT compound and snap-frozen using dry ice. Mesenteric lymph nodes were collected and embedded separately in OCT compound and snap-frozen using dry ice.

### IHC analysis.

A fresh frozen section (5 μm) was fixed in 100% acetone for 10 minutes at room temperature and blocked with 1.5% bovine serum albumin for 30 minutes. The sections were stained with the fluorochrome-conjugated antibodies listed in Table 1 and kept in a cold room overnight. Secondary antibody staining was performed, followed by DAPI staining for nuclei. The section was mounted in 90% glycerol in PBS and visualized and analyzed using a Zeiss LSM 980 confocal microscope (Zeiss) and Fiji ImageJ software.

### Flow cytometry.

After removing Peyer’s patches, the small intestine was cut into small pieces, and lamina propria cells were prepared by removing epithelial cells with 1 mM EDTA for 20 minutes. The cells were then dissociated in collagenase IV medium (1 mg/mL of collagenase with 0.1 mg/mL of DNase I) for 20 minutes after rinsing the cell pellet with PBS. Viable immune cells from the lamina propria were purified through 2 steps using magnetic nanobeads. First, a dead cell removal kit (BioLegend) was used to exclude dead cells, followed by CD45^+^ immune cell enrichment using CD45 nanobeads (BioLegend). To analyze DC subsets in the mouse small intestine, freshly isolated cells were blocked with 2.4G2 (anti-Fc receptor) to prevent nonspecific staining and stained with the fluorochrome-conjugated antibodies listed in Table 1. Cells were collected and analyzed using a cell sorter SH800S (Sony Biotechnology) and FlowJo software (BD Biosciences).

### T1L reovirus infection model.

T1L reovirus was prepared according to the previously described (18). Mice were inoculated perorally with purified reovirus diluted in PBS using a 22-gauge round-tipped needle. All T1L reovirus–treated mice were given the same dose of 1 × 10^8^ PFU of virus and sacrificed for analysis 48 h post-infection.

### Human PBMCs analysis.

Blood samples were collected from patients with CeD and nonceliac donors. The red blood cells were removed before use of PBMCs. PBMCs from patients with CeD (*n* = 4) and nonceliac donors (*n* = 3) were incubated in RPMI medium containing 2 μM HB-230 for 90 minutes. Following incubation, the cells were washed twice with PBS that contained 2% FBS and stained with the antibody cocktail detailed in Table 1. The cells were subsequently collected and analyzed using a cell sorter SH800S (Sony Biotechnology) and FlowJo software (BD Biosciences).

### Statistics.

Statistical comparison groups were performed using the Mann-Whitney *U* test or 2-way ANOVA with multiple comparisons, and assessed using the 2-stage step-up method of Benjamini, Krieger, and Yekutieli; 1-way ANOVA with Tukey’s multiple-comparison test; or 2-tailed Student’s *t* test. Statistical analysis was performed with GraphPad Prism 10. *P* values of less than 0.05 were considered significant.

### Study approval.

Mice studies were conducted in accordance with protocols approved by the APLAC at Stanford University and the Institutional Biosafety Committee and the Institutional Care and Use Committee of the University of Chicago. Blood samples from patients with CeD (Stanford Medicine Outpatient Center in Redwood City) and nonceliac donors (healthy individuals from Stanford Blood Center) were collected with permission from the Stanford IRB (protocol no. 20362).

### Data availability.

Data related to the paper are available from the corresponding author on reasonable request. Values for all data points in graphs are reported in the Supporting Data Values file. 

## Author contributions

FCY, CK, and BJ designed the research and supervised all investigations. FCY, HAB, and HRC performed experiments and analyzed data. CK and FCY wrote the manuscript. MA contributed to establishing the animal model, and NQFB provided blood samples from celiac and patients without celiac disease. All authors provided critical reviews of the manuscript.

## Supplementary Material

Supplemental data

Supplemental video 1

Supporting data values

## Figures and Tables

**Table 1:**
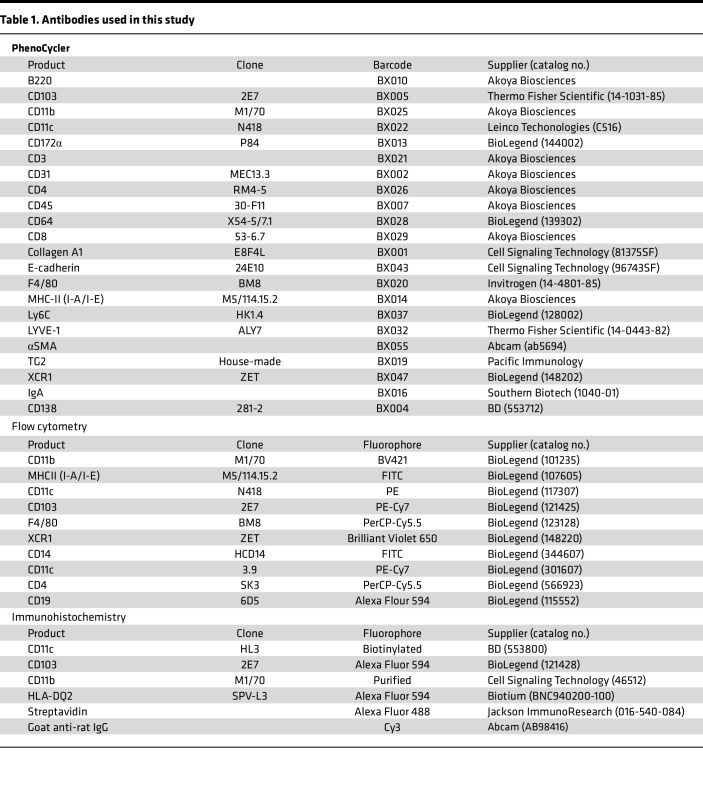
Antibodies used in this study

## Version 1 08/28/2025

In-Press Preview

## Version 2 10/08/2025

Electronic publication
