# Supplementary material for: A dendritic cell population responsible for transglutaminase 2–mediated gluten antigen presentation in celiac disease
Source: JCI Insight. 2025 Aug 28;10(19):e196102. doi: 10.1172/jci.insight.196102 (PMC12513477; doi:10.1172/jci.insight.196102)

SUPPLEMENTAL FILE

A dendritic cell population responsible for transglutaminase 2-mediated gluten antigen presentation in celiac disease

Fu-Chen Yang[1], Harrison A. Besser[1,2,#], Hye Rin Chun[3,#],

Megan Albertelli[4], Nielsen Q. Fernandez-Becker[5], Bana Jabri[3,6], and Chaitan Khosla[1,7,8,*]

[1]Department of Chemistry, Stanford University, Stanford, California, USA

[2]Stanford Medical Scientist Training Program, Stanford University School of Medicine, Stanford, California, USA

[3]Committee on Immunology, University of Chicago, Chicago, Illinois, USA

[4]Department of Comparative Medicine, Stanford University, Stanford, California, USA

[5]Division of Gastroenterology and Hepatology, Department of Medicine, Stanford University, Stanford, California, USA

[6]Department of Medicine, University of Chicago, Chicago, Illinois, USA

of Medicine, Stanford University School of Medicine, Stanford, California, USA

[7]Department of Chemical Engineering, Stanford University, Stanford, California, USA

[8]Sarafan ChEM-H, Stanford University, Stanford, California, USA

[#]H.A.B. and H.R.C. contributed equally to this work.

*Correspondence:

Name: Chaitan Khosla

Address: 290 Jane Stanford Way, Stanford, CA 94305, USA

Telephone: +1(650) 723-6538

E-mail: Khosla@stanford.edu

Supplemental Figure legends

Figure 1. HB-230-labeled active TG2 is observed in the small intestines of WT mice but not in TG2KO.

(A) A schematic illustration of the Swiss-roll preparation of the ileum, distal jejunum, and Peyer's patches in the mouse small intestine. Representative images of Swiss-rolls from WT and TG2KO mice dosed with HB-230 2 h before sacrifice. White arrows in the WT image indicate HB-230 signals within the tissue. (B) WT mice received 10 mg/kg HB-230 orally 24 h before euthanasia. After sacrifice, the small intestine and MLN were collected, embedded, and snap-frozen separately. All images were taken using a Zeiss confocal microscope at 40x magnification.

Figure 2. The percentage of HB-230$^+$ cells among macrophages in mice dosed with HB-230 or PBS.

Small intestinal lamina propria cells were isolated from WT and TG2KO mice after receiving HB-230 or PBS 2 h before sacrifice. Viable CD45$^+$ immune cells were enriched and stained with antibodies. (A) Macrophages were gated on MHCII$^+$F4/80$^+$ cells, and CD103$^+$ DCs were gated on F4/80$^-$MHCII$^+$CD11c$^+$. No changes in overall populations of either cell type were observed in response to HB-230 treatment. (B) Among macrophages, HB-230 was gated based on the PBS control group (WT, n=7; TG2KO, n=7).

Figure 3. Extracellular HB-230 colocalizes with collagen and smooth muscle actin.

WT mice received HB-230 at a dose of 10 mg/kg via oral administration. Two hours after administration, tissues were collected, snap-frozen, and stained with antibodies. Images were captured and analyzed using the PhenoCycler system. (A) (Left) Images of a representative cryosection with extracellular HB-230 overlaid with labeled antibodies against TG2 protein and type I collagen. (Right) Magnified images of the lamina propria (Box 1) and muscularis (Box 2). (B) Extracellular HB-230 overlaid with TG2 protein and anti-PDGFRα antibody near the basement membrane of villi (Box 2). (C) A representative cryosection labeled with HB-230, antibody against TG2 protein, and antibodies against markers of non-immune cell compartments in the small intestine, including epithelial cells (E-cadherin), smooth muscle cells (αSMA), lymphatic vessels (LYVE-1), and endothelial cells (CD31). On the right, % HB-230^{+} cells among epithelial cells and endothelial cells. Each point represents a normalized quantity of E-cad^{+}HB-230^{+} cells in E-cad^{+} cells or $CD31^{+}$HB-230^{+} cells in $CD31^{+}$ cells from 10 villi. For each mouse, two randomly picked regions with 10 villi were analyzed (WT, n=3). All representative images were captured using a confocal microscope at 20x magnification. Data represent mean ± SEM.

Figure 4. Gluten peptides reach MLNs following oral administration but are not presented by $CD103^{+}$ DCs to T cells.

WT mice received 10 mg/kg of HB-298 orally for 2 h before sacrifice. After euthanasia, MLNs were collected, snap-frozen, and stained with anti-CD103 antibody. Some HB-298 is observed

in the MLN post-doing but does not colocalize with $CD103^{+}$ DCs. The image was taken using a confocal microscope at 40x magnification.

Figure 5. Elevated extracellular HB-230 is observed in DR3.DQ2 mice when on an enhanced gluten diet.

WT and DR3.DQ2 mice were given normal chow or specified diets for 14 days prior to receiving 10 mg/kg HB-230 orally. Two hours after administration, tissues were collected, snap-frozen, and stained with antibodies. (A) Elevated extracellular HB-230 was noted at the tips of the villi in DR3.DQ2 mice that received a high-gluten diet for 14 days. Five representative images from 2 to 3 mice were taken using a confocal microscope at 40x magnification. (B) The left image demonstrates that intracellular HB-230 counting using QuPath software (36) to identify $HB\text{-}230^{+}$ cells in each villus. The dot plot shows $HB\text{-}230^{+}$ cells per villus in each cohort (n=10). P values were determined using an unpaired Mann-Whitney two-tailed *t*-test. **, $p < 0.01$. Data represent mean ± SEM.

Figure 6. Analysis of small intestinal samples from WT and DdVil-IL15 mice.

WT and DdVil-IL15 mice were orally administered 10mg/kg HB-230 for 2 h before sacrifice, followed by staining with antibodies. (A) Representative confocal microscopic images of distal and proximal Swiss-rolled small intestine sections of WT or DdVil-IL15 mice 2 h post HB-230 administration (HB-230 in magenta; CD11c in green; nuclei in blue). (B, C) The dot plots show the percentage of $HB230^{+}$ cells within $CD103^{+}CD11b^{+}$ cDC2 (B) and $CD103^{+}XCR1^{+}$ cDC1 (C)

subsets. Statistical significance was determined using one-way ANOVA with Tukey's multiple comparison test; *, $p < 0.05$. Data represent mean ± SEM.

Figure 7. Reovirus infection increased ECM-associated TG2 activity and HB-230 uptake by $CD103^{+}CD11b^{+}$ cDC2.

(A) Experimental schema of the reovirus infection model. WT and TG2KO mice were administered 3×10^{8} PFU of T1L reovirus or PBS via oral gavage 48 h before receiving 10 mg/kg HB-230. Two hours following HB-230 dosing, mice were sacrificed, and the small intestines were collected for analysis. (B) Representative confocal images of distal and proximal Swiss-rolled small intestine sections (HB-230 in magenta; CD11c in green; nuclei in blue). Intestinal lamina propria cells were isolated from T1L-infected mice, followed by staining with antibodies. The dot plot indicates the percentage of $CD11c^{+}MHCII^{+}F4/80^{-}$ DCs (C) and $CD11c^{+}MHCII^{+}F4/80^{+}$ macrophages (D) among total $CD45^{+}$ or HB-$230^{+}CD45^{+}$ cells. $CD103^{+}$ DCs are subcategorized and analyzed for the percentage of $HB230^{+}$ among $CD103^{+}XCR1^{+}$ cDC1 (E) or $CD103^{+}CD11b^{+}$ cDC2 (F). Statistical significance was determined using two-way ANOVA with Turkey's multiple comparison test. (C, D) or one-way ANOVA with Tukey's multiple comparison test (E, F). *, $p < 0.05$; **, $p < 0.01$. Data represent mean ± SEM.

Supplemental video Time-lapsed, live cell imaging of BMDCs exposed to HB-230 for 90 min.

Supplemental Figure 1

A

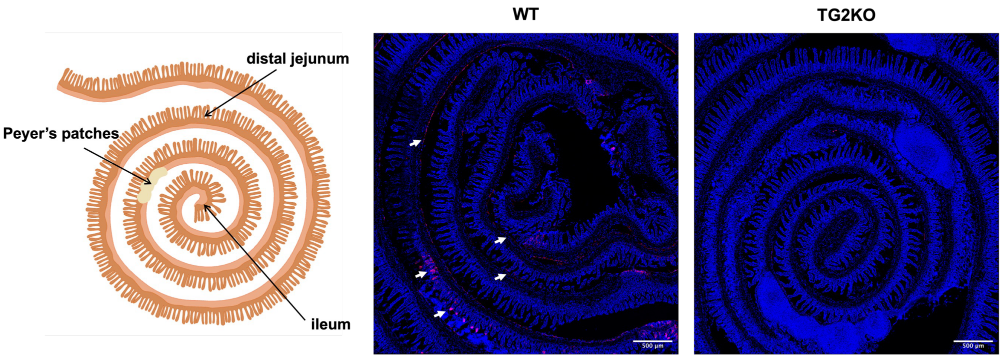

B

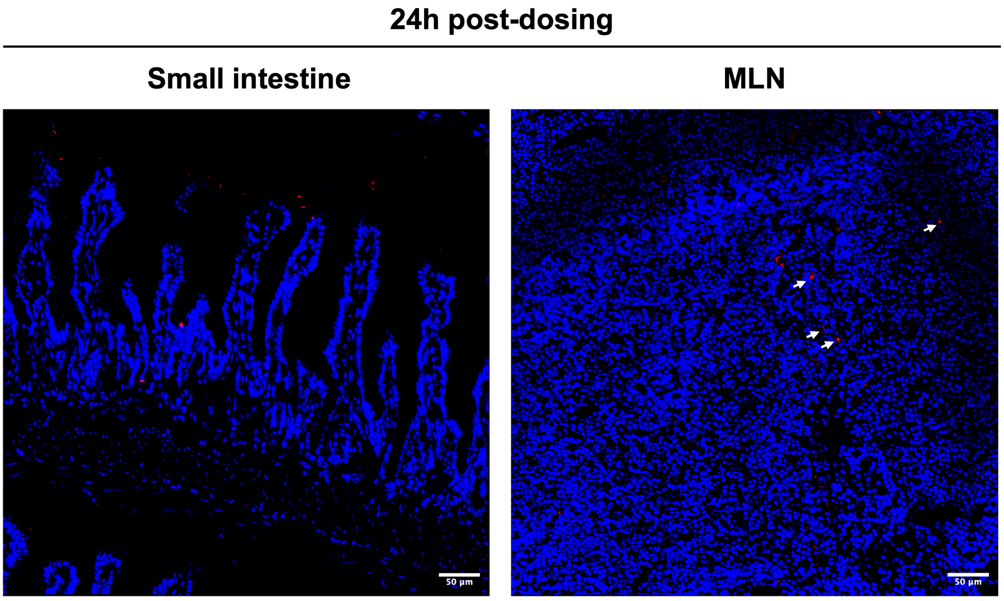

Supplemental Figure 2

A

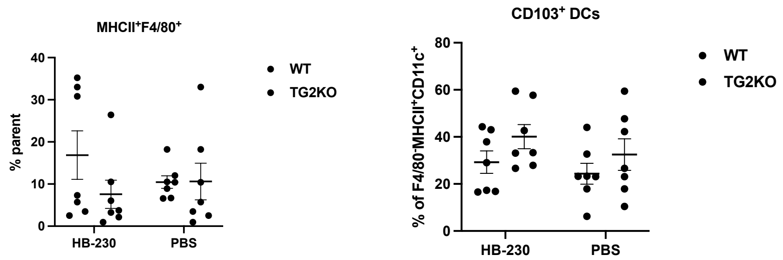

B

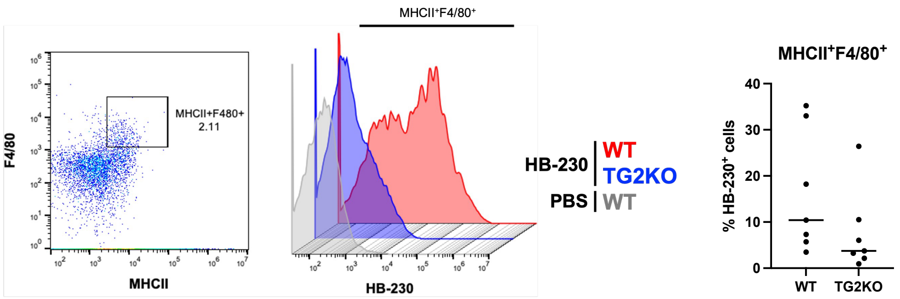

Supplemental Figure 3

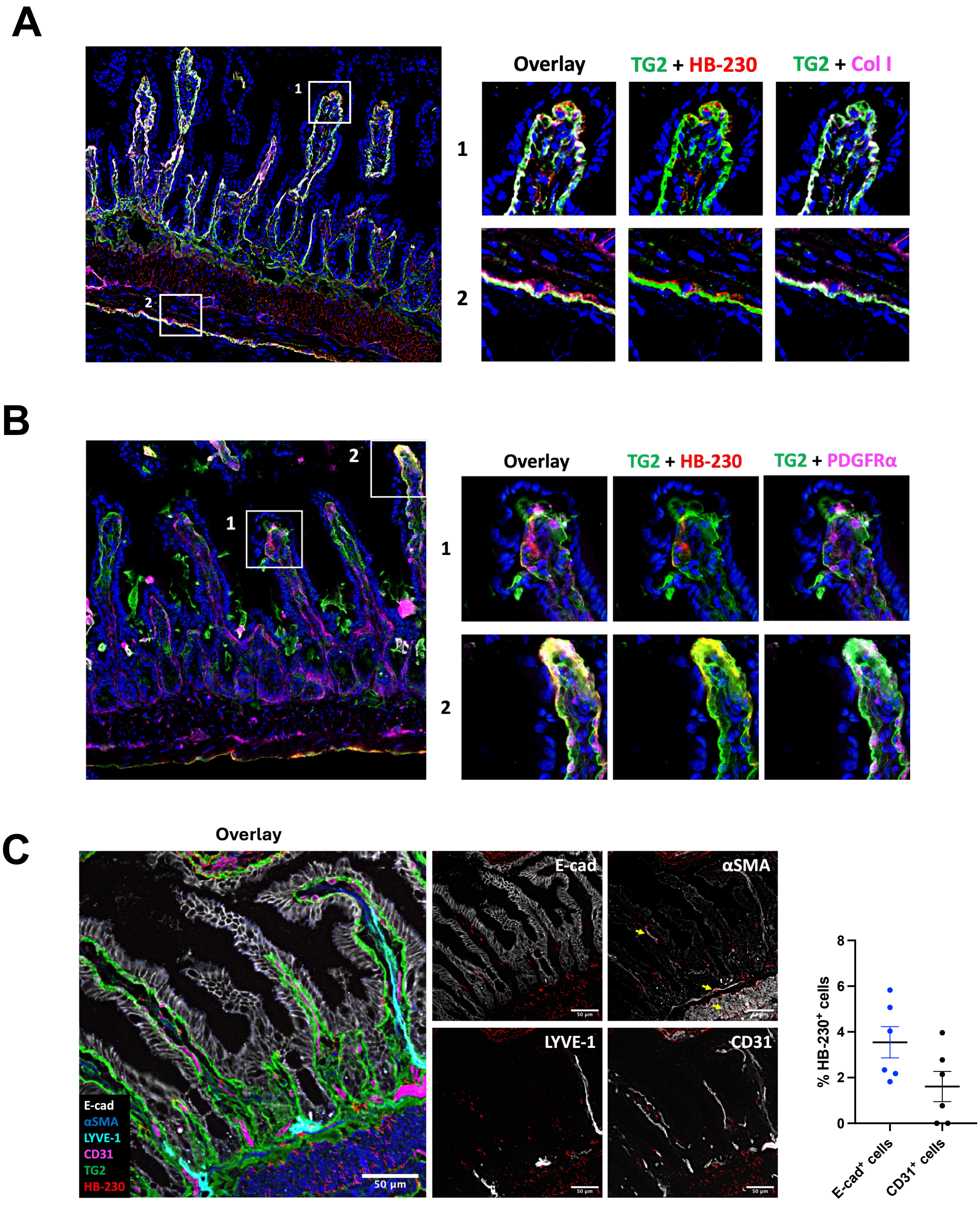

Supplemental Figure 4

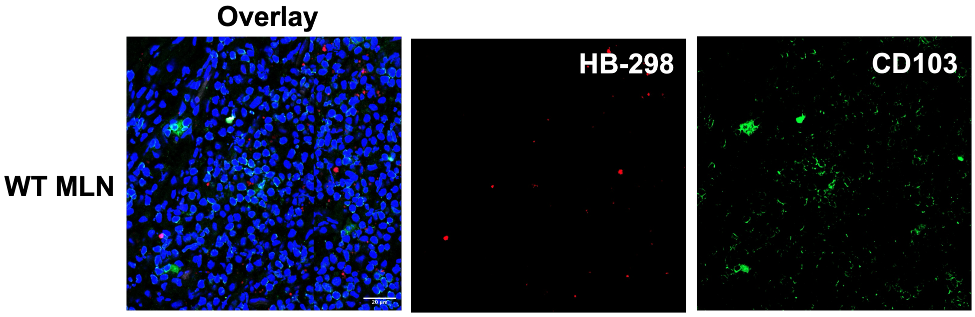

Supplemental Figure 5

A

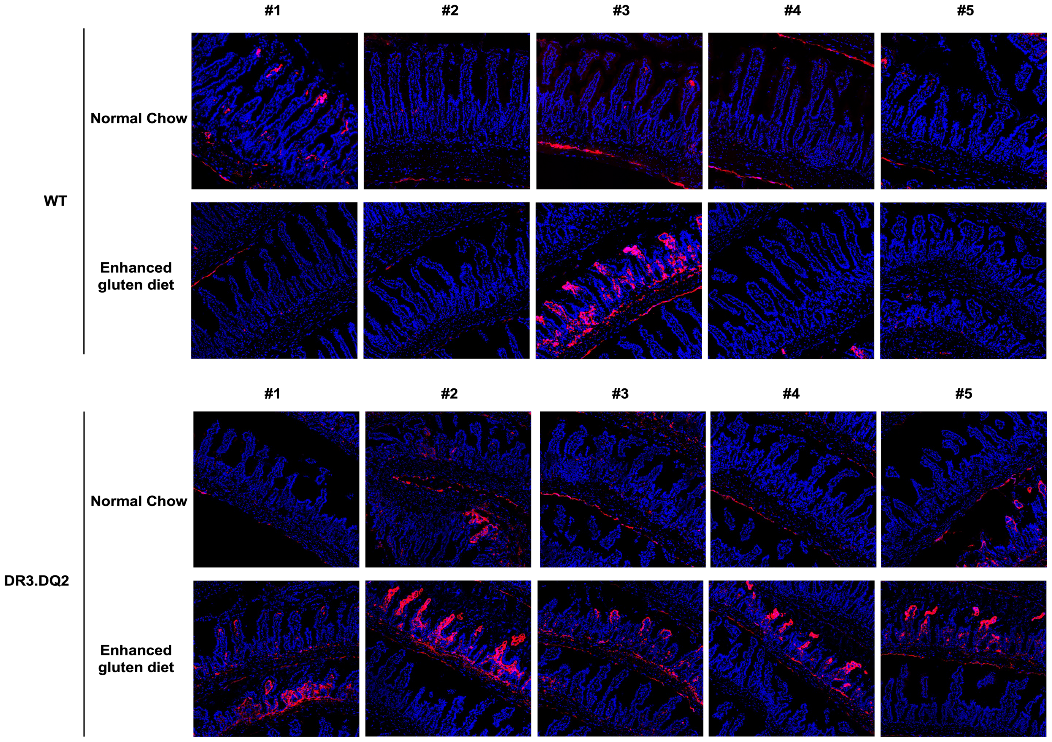

B

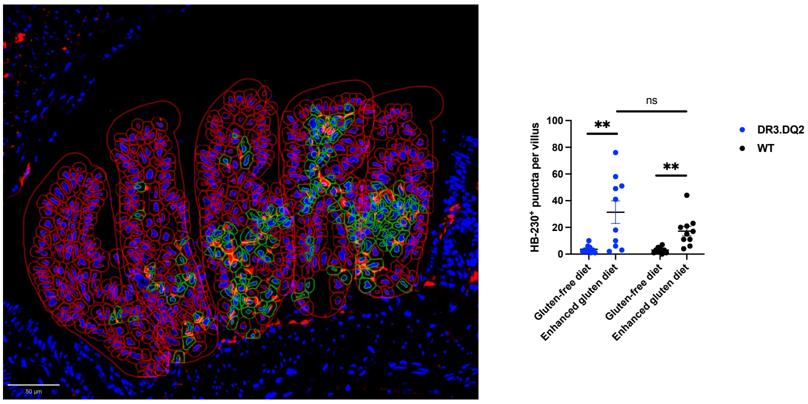

Supplemental Figure 6

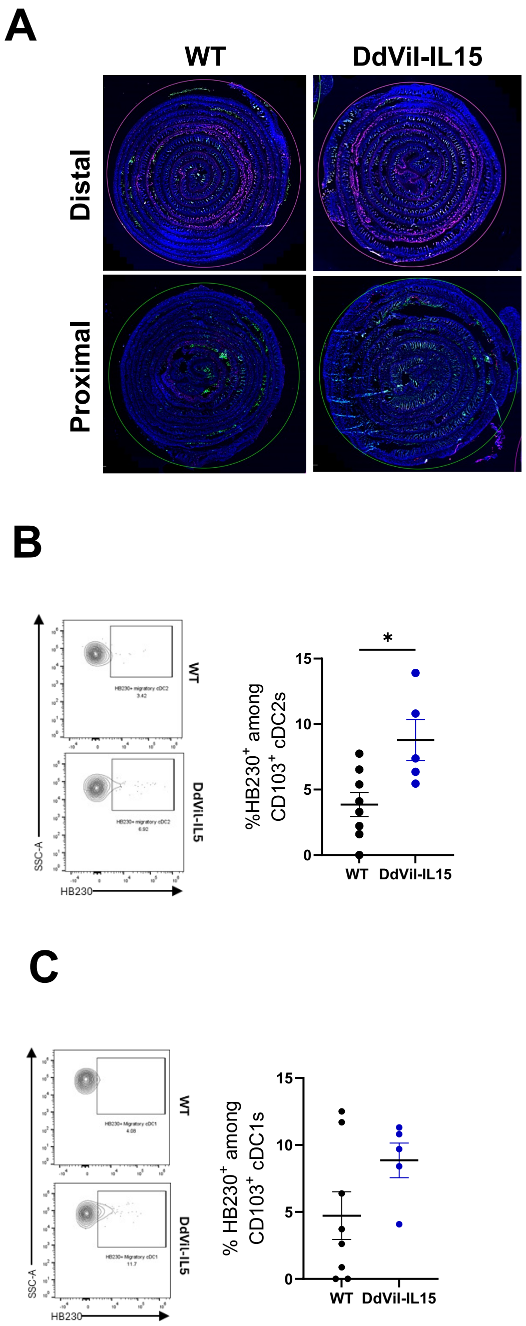

Supplemental Figure 7

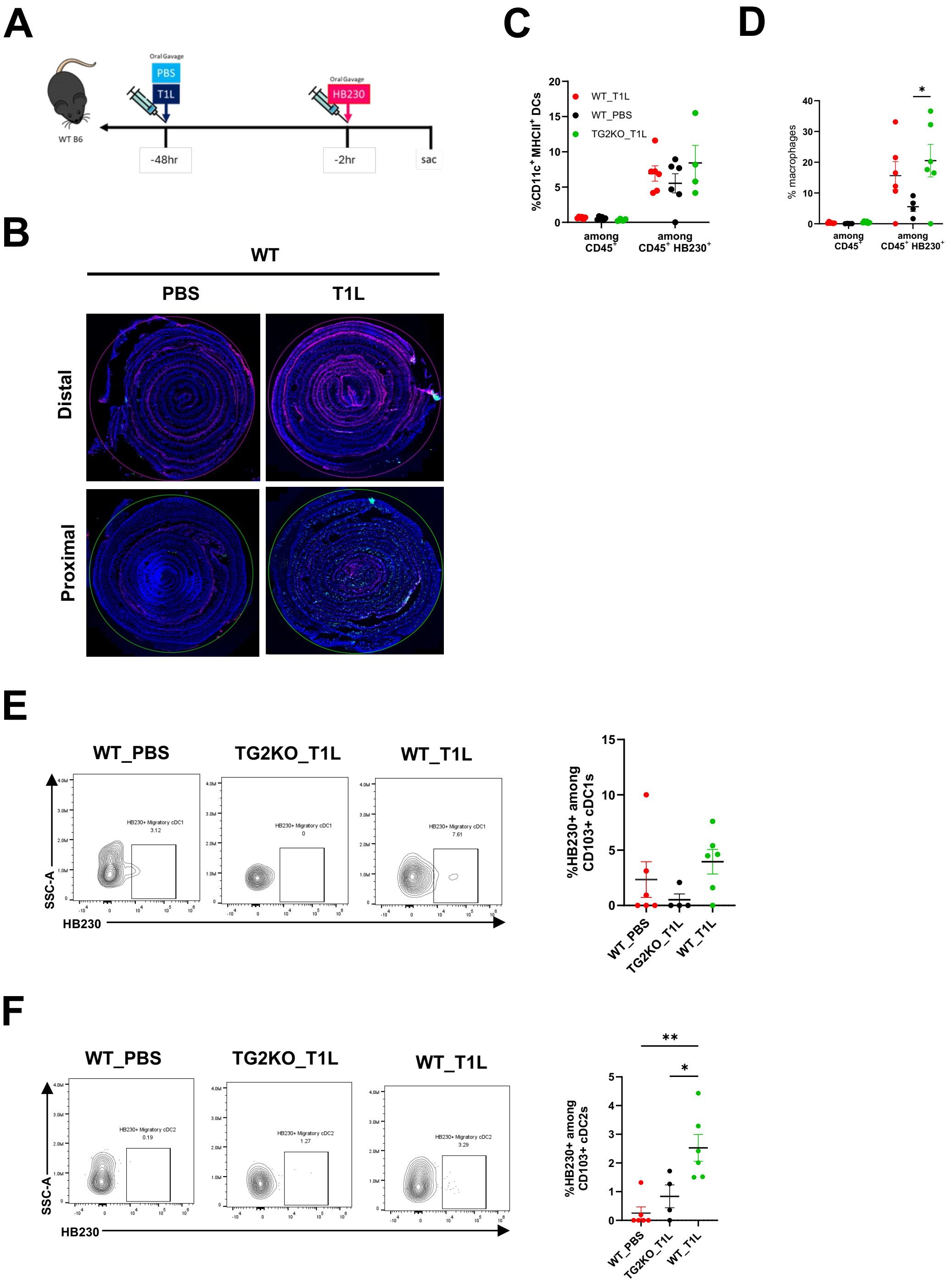

Supplementary video

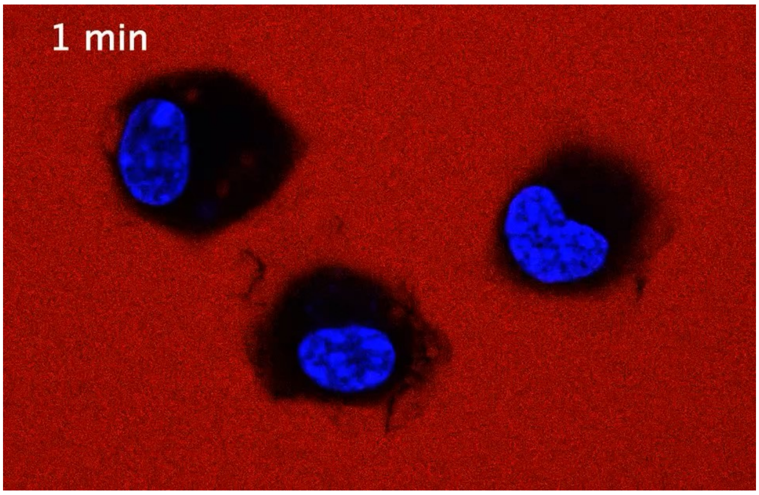

Supplement: Supplemental data [file jciinsight-10-196102-s148.pdf]
